# A role for taurine in mitochondrial function

**DOI:** 10.1186/1423-0127-17-S1-S23

**Published:** 2010-08-24

**Authors:** Svend Høime Hansen, Mogens Larsen Andersen, Claus Cornett, Robert Gradinaru, Niels Grunnet

**Affiliations:** 1 Department of Clinical Biochemistry, 3-01-1, Rigshospitalet, Copenhagen University Hospital, Blegdamsvej 9, DK-2100 Copenhagen, Denmark; 2 Department of Food Science, Faculty of Life Sciences, University of CopenhagenRolighedsvej 30, DK-1958 Frederiksberg C, Denmark; 3 Department of Analytical Chemistry, Faculty of Pharmaceutical Sciences University of Copenhagen, Universitetsparken 2, DK-2100 Copenhagen, Denmark; 4 Department of Chemistry, Al. I. Cuza University of Iasi, 700506 Iasi, Romania; 5 Department of Biomedical Sciences, Faculty of Health Sciences, University of Copenhagen, DK-2200 Copenhagen N, Denmark

## Abstract

The mitochondrial pH gradient across the inner-membrane is stabilised by buffering of the matrix. A low-molecular mass buffer compound has to be localised in the matrix to maintain its alkaline pH value. Taurine is found ubiquitously in animal cells with concentrations in the millimolar range and its pKa value is determined to 9.0 (25°C) and 8.6 (37°C), respectively. Localisation of such a low-molecular buffer in the mitochondrial matrix, transforms the matrix into a biochemical reaction chamber for the important matrix-localised enzyme systems. Three acyl-CoA dehydrogenase enzymes, which are pivotal for beta-oxidation of fatty acids, are demonstrated to have optimal activity in a taurine buffer. By application of the model presented, taurine depletion caused by hyperglycemia could provide a link between mitochondrial dysfunction and diabetes.

## Background

### Intracellular localisation of taurine

Taurine has recently been proposed to have an important role in mitochondria in animal cells [1]. The hypothesis is based on the fact that taurine is found in very high concentrations in oxidative tissue, but in lower concentrations in glycolytic tissue. The studies on the contents of porcine muscle tissue determined that taurine is found in high concentrations of 15-20 µmol/g in the oxidative porcine muscle tissue, but only 1-3 µmol/g in the low-oxidative glycolytic muscles [1,3]. Studies on muscles from horse and human have demonstrated that taurine is preferentially localised in oxidative muscle fibres [4,5]. Across the cell membrane, a Na+- taurine symporter maintains a taurine gradient, whereas an osmo-sensitive channel regulates efflux of taurine (for reviews on taurine uptake, volume sensitive release, transporter systems and the involvement in osmoregulation, see [6,7]). If taurine is associated with mitochondria and a taurine transporter system exist across the mitochondrial membranes, the higher taurine content found in oxidative tissue can easily be explained by a simple two-compartment model due to the fact that oxidative tissue is more mitochondria-rich than glycolytic tissue [1].

A study [8] on intracellular compartmentation applied analytical NMR spectroscopy for studying the low-molecular composition of isolated mitochondria compared to intact rat heart tissue. When comparing the intensities in the shown spectra in [8] of intact tissue, tissue extract, isolated intact mitochondria and mitochondria extract, it is evident that the only low-molecular compounds found in substantial concentrations inside the mitochondria are taurine and lipids. However, as sample preparation retaining the low-molecular fraction is very difficult in such studies, the results can only be evaluated qualitatively, but taurine seems to have an important role in mitochondria. Further support is obtained from the results of several immunocytochemical localization experiments of taurine [9,10]. These studies have clearly demonstrated a preferential localization of taurine in the mitochondria compared with the cellular cytosol through immunogold labelling of taurine and application of electron microscopy.

A very recent report [11] on the taurine concentration found about 70 nmol/(mg protein) in mitochondria isolated from rat heart. Mitochondrial protein mass has been reported to represent about 30% of the total mitochondrial mass [12], so 70 nmol/(mg protein) corresponds to about 20-30 µmol/g wet tissue, and in the mitochondrial water phase a taurine concentration of about 30-40 mmol/l.

Taurine has also been demonstrated to be a constituent of modified uridine residues in mitochondrial tRNA [13]. It was shown that the nucleotides were synthesized *in-vitro* in the mitochondria when supplying taurine to the cell incubation medium. Furthermore, the study reported that taurine was taken up by isolated mitochondria, although the mitochondrial taurine transporter has not yet been identified. As mitochondrial tRNA is synthesized in the mitochondrial matrix, subsequent processing and modification is expected to be performed inside the mitochondrial matrix as well, thus demonstrating the matrix existence of taurine. The study also showed that taurine-modified uridines were lacking in mutated mitochondrial tRNAs associated with mitochondrial diseases. Due to the role in mitochondrial tRNA, taurine has been suggested an important role directly in the translation and expression of the mitochondrial respiratory proteins. This line of argument can be found presented elsewhere [14].

### Mitochondrial oxidation and the chemiosmotic theory

The chemiosmotic theory proposed by Peter Mitchell in 1961 [15] and refined during the 1960s is today considered as the theoretical basis for understanding of the oxidative processes and ATP production in the mitochondria [16,17].

The chemiosmotic theory can be formulated as follows: ATP production by ATP synthase is driven by a proton flux across the mitochondrial inner-membrane. The the chemical potential **Δμ** for the proton flux across the membrane, can be expressed as a sum of two contributions:

Δμ = FΔΨ – 2.303 RT ΔpH

The term **FΔΨ** is the electrical membrane potential across the inner-membrane multiplied by the Faraday constant **F**. The contribution **2.303 RT ΔpH** represents the free energy difference associated with the existence of a pH gradient across the membrane.

The amount of protons forming the proton gradient can be considered as temporary proton reservoir to dampen transients and fluctuations in the subsequent proton flux. Actually, the use of traditional water towers to create a stable water supply could be compared with the role of the proton gradient.

The existence of the pH gradient was demonstrated experimentally in the late 1960s and 1970s by different techniques like indicator compounds or microelectrodes [18]. Later, application of fluorescent protein techniques on mammalian cells has confirmed the existence of the pH gradient by reporting a mildly alkaline pH value for the mitochondrial matrix of 7.9-8.4 and for the cytosol 7.2-7.4 [19,20].

### Buffering capacity of the mitochondria

The mitochondrial pH buffering capacities for outer and inner compartments stabilise the pH gradient. However, except for the original studies by Mitchell and Moyle [16,17,21], few studies or presentations, if any, can be found on the relationship between the pH gradient and the mitochondrial buffering capacities.

Mitchell and Moyle determined the pH buffering capacities of rat liver mitochondria as a function of pH [21]. In the pH range from 7.5-8.5, the total buffering capacity was determined to be about 40-50 µmol H^+^/g mitochondrial protein, which was divided into the contributions of outer mitochondria buffering capacity (30-40 µmol H^+^/g mitochondrial protein) and inner mitochondrial buffering capacity (10-20 µmol H^+^/g mitochondrial protein). Mitochondrial protein mass has been reported to represent about 30% of the total mitochondrial mass [12].

However, examination of the tissue preparation reported in the classical study by Mitchell and Moyle (see Figure 6 and 7 in [21]) reveal that any water-soluble low-molecular compounds localised inside the mitochondria is likely to have been washed away before performing the pH titrations. Nothing is mentioned about low-molecular compounds contributing to the pH buffering, i.e. any mitochondrial-localised low-molecular compound like taurine in high concentrations has probably not contributed to the determined buffering capacities.

In addition, it is relevant to compare with some classical studies on fish skeletal muscles [23] or on mammalian skeletal muscle [24], as these studies on tissue fractionation with subsequent titrations found that about two thirds of the buffering capacity, even at pH 8.0, is due to low-molecular mass compounds.

**Figure 1:**
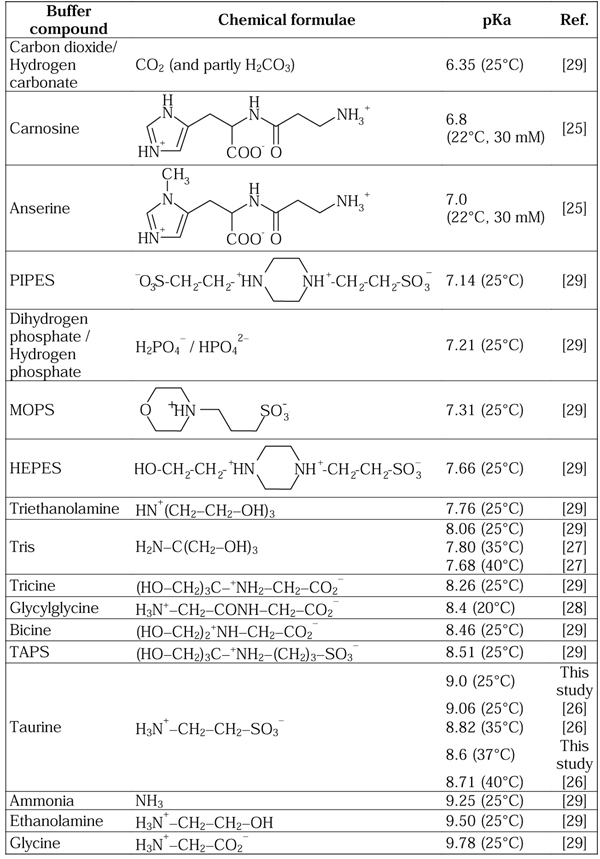
**Examples of buffer compounds divided into accepted physiological buffers and synthetic buffers used traditionally in biological and biochemical research.** The buffers are listed with corresponding pKa values. Except for the pKa values for carnosine, anserine and taurine, no reports are available on the ionic strength used for the pKa determination.

The well-known physiological buffer systems, carbon dioxide / hydrogen carbonate and hydrogen phosphate / dihydrogen phosphate, are possibly the most obvious low-molecular candidates for mitochondrial buffering. However, as seen from the pKa values in Figure 1, these buffer systems can provide acceptable buffering in the compartment controlling the low pH of the pH gradient (pH 7.0-7.5), whereas their buffering capacity seems inadequate in a mildly alkaline compartment like the mitochondrial matrix (pH 7.9-8.5).

### Biological buffers

In porcine muscle tissue, the histidine-related compounds carnosine and anserine identified as buffers can be found in the concentration range of 10-15 µmol/g in muscles with low oxidative capacity and only 1-3 µmol/g in the oxidative muscles [1,3]. These compounds are considered as glycolytic buffers and thus to be found in the cytosol. In addition carnosine and anserine will not be able to provide sufficient buffering capacity at pH 8.0 and above with pKa values about 7.0 (see Figure 1).

Considering low-molecular mass components, only a limited number of compounds are traditionally considered as physiological buffers. Besides, only a limited number of synthetic buffers are traditionally used in cell biology and biochemistry. An overview of such synthetic biochemical research and physiological buffers (including taurine) is presented in Figure 1. In spite of the non-physiological origin of many of the buffers, they have been used in many biochemical and cell biological studies with subsequent physiological conclusions. It should be noticed that HEPES and PIPES contain the amino ethane sulphonate structural element of taurine. In many lists of buffers to be found elsewhere [28,29] or from laboratory chemical vendors, no or very few compounds with a pKa of about 8-9 can be found. However, in Figure 1 we have included Taurine, the commonly used Tris and Glycylglycine, as well as the rarely used Tricine, Bicine and TAPS.

## Results and discussion

### Buffering properties of taurine

**Figure 2:**
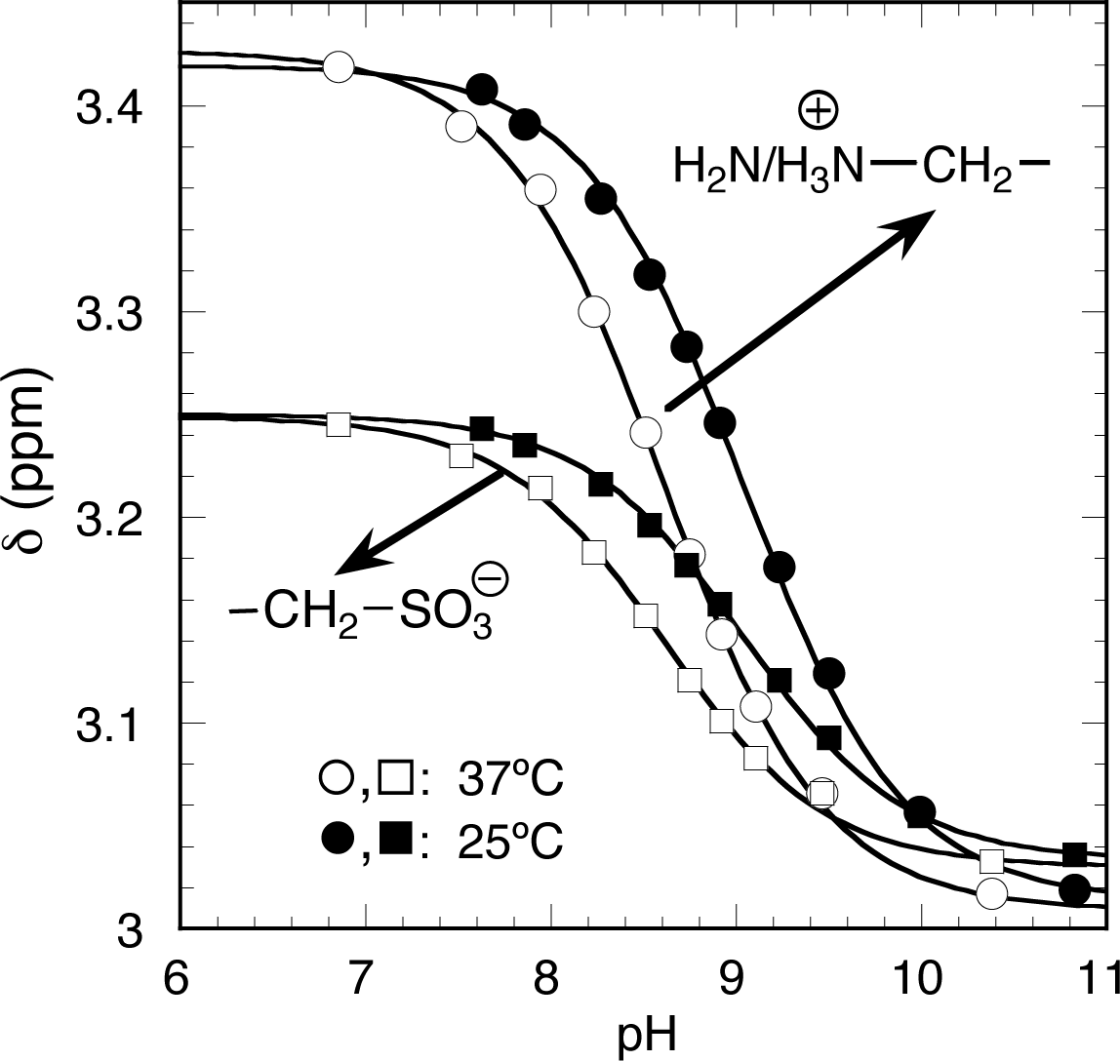
**pH dependence of the ^1^H NMR-spectra of taurine at 25°C and 37°C.** pH dependence of the chemical shift (⊠) at 25°C (solid triangles and squares) and 37°C (open triangles and squares) of the signals. The lines through the data points were obtained from fits using the pH equation. The estimated pKa values are 9.0 (25°C) and 8.6 (37°C).

The sulphonate group in taurine with a pKa value about 1.5 (at 25°C) is negatively charged at all physiological pH values, whereas we have determined the pKa value for the amino group mimicking physiological conditions with regard to temperature and intracellular ionic osmolarity, i.e., 37°C, 20 mM taurine and total osmolarity of approximately 300 mOsmol/l. The taurine titration was monitored by ^1^H NMR spectroscopy (Figure 2). The chemical shift (⊠) and the coupling of the taurine methylene hydrogens depend on the ionisation of its amino groups and thus on the pH in the range 7-10.

From the data of Figure 2, the pKa value for the taurine amino group was estimated to 9.0 and 8.6 at 25°C and 37°C, respectively. Thus, the pKa value of taurine at 37°C is within the physiological relevant pH range. As both values are in good agreement with values reported for zero ionic strength [26]: pKa=9.06 (25°C), pKa=8.82 (35°C), and pKa=8.71 (40°C), no major changes of the pKa can be expected in the intracellular environment.

The pKa value of taurine means that mitochondrial-localised taurine would contribute significantly to the mitochondrial pH buffering capacity in the pH range from 7.5-8.5. A taurine concentration of about 70 µmol/(g protein) in mitochondria [11] represents a buffering capacity of about 15-40 (µmol H^+)^/(g protein) with the maximum value at pH=pKa. In the classical study by Mitchell and Moyle, the lowest values for buffering capacity were about 40 (µmol H^+^)/(g protein) observed in the pH range from 7.0-8.5 (compare again with Figure 6 and 7 in [21]). Obviously the values are similar, but to draw the correct conclusion the mitochondrial pH buffering capacities should be determined again in a study with controlled concentrations of taurine.

It should be noted that the concentrations of the compounds contributing to the mitochondrial pH buffering capacity need to be in balance with the capacity and regulation of the proton pumping due to the electron transport chain, ATP-synthase and the proton leak through uncoupling proteins. If the pH buffering capacity is too low, the proton pumping could lead to excessive proton leak or even cause that the matrix becomes too alkaline with subsequent mitochondrial damage.

It is reasonable to compare the pH buffering capacity with the proton pumping turnover in active mitochondria. Values for oxygen consumption can be found to be about 100-400 (μmol O)/(min∙g protein) [30]. As each oxygen equivalent pumps 10 protons, a proton turnover of 1000-4000 (μmol H^+^)/(min∙g protein) can be expected. Although a proton buffer capacity of 15-40 (µmol H^+^)/(g protein) seems small, it means that the pH buffering capacity can compensate for transients in oxygen supply of about 0.1-1 s, and thus will provide a more stable ATP production.

### The mitochondrial matrix as biochemical reaction chamber

Stabilising the pH gradient by the presence of a buffer compound like taurine in the mitochondrial matrix transforms the matrix into a biochemical reaction chamber with a slightly alkaline pH 8.0-8.5. It is well established that several important biochemical processes occur inside the mitochondrial matrix, e.g., all the reactions in the citric acid cycle, fatty acid oxidation and some of the reactions in the urea cycle.

In experimental studies of enzymatic reactions, a number of different buffers are routinely used for controlling pH drifts resulting from the enzyme activity. Studies on mitochondrial enzymes have traditionally used non-physiological biological buffers like Tris, triethanolamine, MOPS or HEPES. This fact can affect the enzyme activities, pH / activity profiles and pH optima.

Previously we have studied the pH activity profile of isocitrate dehydrogenase from the tricarboxylic acid cycle [1]. The influence of the temperature and the nature of the buffer on the enzyme activity were studied in two buffer systems (Tris and taurine). It was found that that the pH optimum at 37°C is in the pH range 8.0-8.5 for both buffers. This pH optimum coincides with the expected pH in the mitochondrial matrix and with the maximal buffering capacity of taurine as buffer (pKa=8.6 at 37°C).

**Figure 3:**
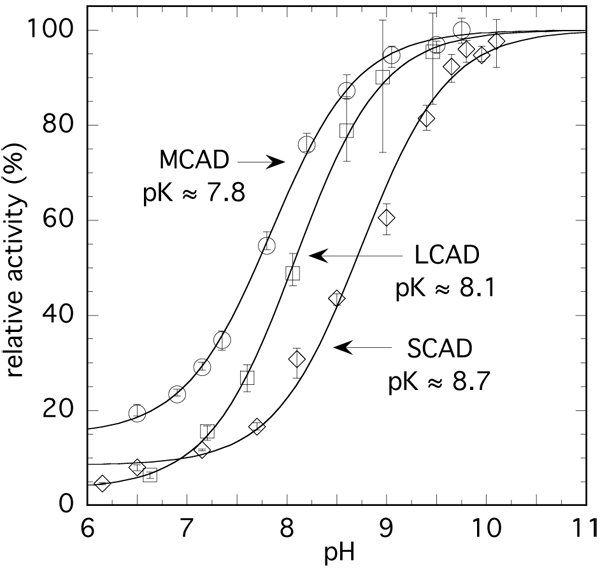
**pH dependence of the activity of acyl-CoA dehydrogenases in taurine buffer.** Conditions: [taurine] = 47 mM, [C_8_-CoA] = 100 µM, [C_12_-CoA] = 35 µM, [C_4_-CoA] = 100 µM at 37°C (substrate concentrations are ≥5•Km). Enzyme concentration in the assays was 60 nM. Enzyme activities were estimated using the ferricenium assay [33] and are normalised for the sake of comparison. The activities values (turnover) extrapolated to low and high pH are 560 and 3800 min^-1^ for MCAD; 85 and 2400 min^-1^ for LCAD and 160 and 2050 min^-1^ for SCAD respectively. The pK values are derived from the curve fits. The data points are the average of 3-4 individual measurements and the vertical bars indicate the scatter where appropriate.

The acyl-CoA dehydrogenases (ACADs) control the beta-oxidation of fatty acids in animal cells and are important mitochondrial enzymes involved in fatty acid degradation. These enzymes exhibit a comparatively low activity at pH <7 that increases markedly with pH [32]. This dependence reflects apparent pK values of 8-9, also depending on the specific ACAD (e.g. medium-chain acyl-CoA dehydrogenase [MCAD], long-chain acyl-CoA dehydrogenase [LCAD], or short-chain acyl-CoA dehydrogenase [SCAD]), on the chain length of the substrate, and on the composition of the buffer system [32]. It is important to note that the specific activity can increase by a factor >10 on going from the low pH minimum to the high pH values [32]. However, reported pH/activity studies with ACADs used in general Tris buffer at 25°C and in the presence of a constant concentration of electrolyte (250 mM KCl) [32], conditions that differ substantially from those in the cell. We have thus reinvestigated some of these parameters at 37°C in taurine buffer. Figure 3 shows activity/pH profiles for MCAD, LCAD and SCAD obtained using the ferricenium assay [33]. The activities of these enzymes have rather low values at pH <7 that increase up to 20-fold at pH ≥ 9 reflecting apparent pK values of 7.8-8.7. These apparent pK values compare well with those reported earlier [32], the minor differences being attributed to the variation of the ionic strength. In a general sense, these data demonstrate that fatty acid metabolism requires sufficient pH buffering of the matrix, as the beta-oxidation would be impaired if acidification occurs.

A metabolic regulation argument for the importance of pH buffering can now be based on the fact that the acetyl-CoA produced by beta-oxidation is oxidized to carbon dioxide in the tricarboxylic acid cycle. Production of carbon dioxide and an excessive saturation of its removal processes would induce a pH lowering, and thus cause lower lipid oxidation. Consequently, the carbon dioxide production and subsequent pH control of the mitochondrial matrix is vital for understanding metabolic regulation.

### Mitochondrial preservation and respiratory measurements

In experiments on preservation of oxidative phosphorylation when isolating mitochondria from muscle and heart tissue, it was found that taurine was the most efficient stabilizer of the compounds studied [34]. An ex-vivo study on bovine eyes clearly demonstrated that reperfusion with a taurine solution (1 mM) protected the mitochondria from swelling and subsequent degradation [35]. Other studies on ischemia and reperfusion using taurine solutions have shown clear beneficial effects of a taurine pre-treatment [36]. These studies demonstrate that high extracellular taurine concentrations preserve mitochondrial function and thus prevent the damaging oxidative burst often observed in reperfusion. A possible explanation to these results is as follows: The extracellular taurine prevents taurine from being washed out from from the mitochondria. Consequently, the taurine concentration is kept at an adequate concentration in the mitochondrial matrix to have correct function of the proton pumping, so protection is obtained from the risk that excessive proton pumping will make the mitochondrial matrix too alkaline causing mitochondrial degradation.

Besides addition of taurine in media for mitochondrial preservation, taurine has also been included in media recommended for respiratory measurements [37]. However, an artificial buffer like HEPES is also included in the media. Traditionally respiratory measurements have been performed in media containing artificial buffers like HEPES or Tris. In Table 1, we compare data for oxygen uptake at 30°C in permeabilised rat primary myotubes using either Tris or Taurine as buffer compounds. The results show no difference between the two compounds, i.e., taurine can be used as a biological buffer in cellular systems.

**Table 1:** Oxygen uptake in permeabilised myotubes either in Tris or Taurine buffer.

Buffer	Oxygen uptake, nmol/(min mg protein)		RCR	
	Mean	S.E.M.	Mean	S.E.M.
		
**Tris**	20.3	5.9	1.94	0.39
**Taurine**	19.7	2.6	1.89	0.40

Oxygen uptake was measured with 2 mM malate, 2 mM pyruvate and 2 mM ADP.

RCR is the fold increase in oxygen uptake by addition of 2 mM ADP to incubations with 2 mM malate and 2 mM pyruvate.

## Conclusions and perspectives

The results obtained are all in accordance with the hypothesis of taurine as mitochondrial matrix buffer. The obtained pKa value for taurine is ideal for controlling the metabolic activity of the ACAD enzymes due to the obtained very steep activity increase in slightly alkaline environment. However, obviously additional research is necessary to fully understand the involvement of taurine in mitochondrial function, e.g. the mitochondrial uptake system for taurine need to be characterised.

It should be noticed that the model presented with taurine as proton reservoir in the mitochondrial matrix gives an immediate way to explain some of the mitochondrial observations observed in taurine transporter knockout mice [38]. The decreased exercise capacity can be ascribed to reduced proton buffering capacity in the mitochondria. However, the retained cardial function and almost normal physiological function is to be expected, as the cardial function can be interpreted as a steady state condition with regard to proton pumping and thus not depending on proton buffering. An alternative interpretation could be based on developmental differencies of the mitochondria caused by the taurine transporter knockout.

### Perspectives on mitochondrial dysfunction and diabetes

When ascribing an important role as mitochondrial matrix pH buffer to taurine, consequently, depletion of intracellular taurine will be associated directly with mitochondrial dysfunction. Diabetes and the metabolic syndrome are the most obvious such examples, as intracellular accumulation of lipids, carbohydrates and polyols are observed, which causes disturbances in the osmoregulation and implies taurine depletion [31]. However, the hypothesis lacked a causal implication between taurine deficiency and biochemical function. Mitochondrial dysfunction caused by insufficient pH buffering of the matrix could be the missing link.

Recent viewpoints have associated diabetic complications as well as the impaired insulin secretion and dyslipidemia in type 2 diabetes with mitochondrial dysfunction as unifying hypothesis [39,40]. By applying the presented mitochondrial matrix-buffering hypothesis, the association from hyperglycemia and lipid accumulation to mitochondrial dysfunction can be provided: Intracellular accumulation of lipids, carbohydrates and polyols implies taurine depletion [31], which reduces the mitochondrial matrix-buffering capacity causing impaired oxidation, i.e., mitochondrial dysfunction. Finally, several reports have demonstrated that taurine is necessary for normal insulin secretion from fetal rat islets [41]. Taurine could be interpreted as being necessary for mitochondrial development in the islets. Consequently, interpreting taurine as mitochondrial matrix buffer could improve the understanding of the clinical alterations observed in diabetes.

## Methods

### Titration of taurine

Titration of the amino group in taurine was performed using a solution of 20 mM taurine, 20 mM HCl, 120 mM KCl and subsequent additions of 20 mM KOH. The NMR spectra were acquired using a Bruker Avance 400 WB operating at 400.13 MHz for ^1^H and at 310.2 K (37°C) and at 298.2 K (25°C). A one-dimensional NOESY (Nuclear Overhauser Spectroscopy) sequence was used for suppression of the water signal (the first increment of a 2D NOESY experiment). Spectral simulation and full lineshape regression analysis were performed on all spectra using the gNMR software (Adept Scientific, Hertfordshire, United Kingdom) to obtain chemical shift and coupling constant information. For chemical shift scale reference a small amount of 2,2-dimethyl-2-silapentane-5-sulphonate, sodium salt was added. Immediately before each spectrum was acquired, the sample was thermostated at experiment temperature, and pH was determined by means of a Radiometer PHM 64 research pH meter and a Lazar Research Labs 1113R combination pH electrode. As the NMR spectra typically required 30-60 minutes acquisition time the electrode was calibrated before each measurement using appropriate pH standards from Metrohm.

### Determination of pH profiles of CAD enzymes

Acyl-CoA dehydrogenases. Activity of ACADs was assessed with the ferricenium assay [33] at 37°C in a final volume of 1 ml and following the decrease in absorbance at 300 nm using a UVIKON 933 spectrophotometer. Acyl-CoAs were prepared [42] and purified by preparative HPLC. Acyl-CoA substrate concentrations were determined using ε_260_ = 15.4 mM^-1^cm^-1^ in 5 mM potassium phosphate buffer, pH 7.8. MCAD [43], SCAD and LCAD [44] were obtained as described in the references. Solutions of ferricenium hexafluorophosphate (Aldrich) were prepared freshly and standardized spectrophotometrically in 10 mM HCl, using ε_617_ = 0.41 mM^-1^cm^-1^. For the assay in 47 mM taurine buffer containing 200 µM ferricenium hexafluorophosphate, the final concentrations of the substrates C_8_-CoA (100 µM for MCAD) or C_12_-CoA (35 µM for LCAD) were selected as to be ≥ 5•K_m_. The concentrations of the ACAD enzymes were 60 nM. Data points are the average of 3-4 determinations. The activities were normalised relative to the observed maximal enzyme activity.

Data analysis, secondary plots and fitting routines were done with KaleidaGraph (Synergy Software, Reading, Pennsylvania; USA) and SigmaPlot (SPSS Inc., Chicago, Illinois, USA).

### Oxygen uptake in permeabilised myotubes

Primary rat myotube cultures were prepared as described [45] and permeabilized with 50 µg/ml saponine for 30 min.

The permeabilized cells were scraped off the culture dish, and oxygen uptake was measured with a Clark-type electrode at 30°C. The cell organelles are more stable at 30°C than 37°C.

Tris buffer for determination of oxygen uptake:

5 mM MgCl_2_, 6 H2O (Fluka 63072), 60 mM KCl (AppliChem A1039), 100 mM Mannitol (Mannit no. 63560), 10 mM KH_2_PO_4_ (Fluka 60220), 0,5 mM Na_2_EDTA, 2 H_2_O (Fluka 03677), 60 mM Tris-HCl (Sigma T-3253), 17 mg/ml Phenol red (Sigma P-5530). pH was adjusted to 7.2 at 30°C with potassium hydroxide.

Taurine buffer for determination of oxygen uptake:

5 mM MgCl_2_, 6 H_2_O (Fluka 63072), 60 mM KCl (AppliChem A1039), 100 mM Mannitol (Mannit art. 5982), 10 mM KH_2_PO_4_ (Fluka 60220), 0,5 mM Na_2_EDTA, 2 H_2_O (Fluka 03677), 60 mM Taurine (Sigma T-0625), 17 mg/ml Phenol red (Sigma P-5530). pH were adjusted to 7.2 at 30°C with potassium hydroxide.

## Authors’ contributions

Svend Høime Hansen has developed the concepts and the hypothesis presented. Mogens L. Andersen contributed to the development of the model through critical discussions. Claus Cornett designed and performed the NMR spectroscopic work and associated data handling. Robert Gradinaru has contributed with expert knowledge on the acyl-CoA dehydrogenase enzyme systems and performed the related experiments. Niels Grunnet performed and analysed the oxygen consumption by the permeabilised myotubes.

## Competing interests

The authors declare that they have no competing interests.
